# Ebola Virus GP Activates Endothelial Cells via Host Cytoskeletal Signaling Factors

**DOI:** 10.3390/v14010142

**Published:** 2022-01-13

**Authors:** Benedicte Mpia Moni, Yasuteru Sakurai, Jiro Yasuda

**Affiliations:** 1 Department of Emerging Infectious Diseases, Institute of Tropical Medicine, Nagasaki University, Nagasaki 852-8523, Japan; mpiaben@yahoo.fr (B.M.M.); ysakurai@nagasaki-u.ac.jp (Y.S.); 2 Program for Nurturing Global Leaders in Tropical and Emerging Communicable Diseases, Graduate School of Biomedical Sciences, Nagasaki University, Nagasaki 852-8523, Japan; 3 Department of Virology, National Institute for Biomedical Research, Kinshasa, Democratic Republic of the Congo; 4 Department of Biology, Faculty of Sciences, University of Kinshasa, Kinshasa, Democratic Republic of the Congo; 5 National Research Center for the Control and Prevention of Infectious Diseases, Nagasaki University, Nagasaki 852-8523, Japan

**Keywords:** Ebola GP, ICAM-1, virus–host interactions, cytoskeletal signaling, focal adhesion kinase, TNF-α, host factor, antivirals, micropinocytosis, Rho signaling pathway

## Abstract

Ebola virus disease (EVD) is a lethal disease caused by the highly pathogenic Ebola virus (EBOV), and its major symptoms in severe cases include vascular leakage and hemorrhage. These symptoms are caused by abnormal activation and disruption of endothelial cells (ECs) whose mediators include EBOV glycoprotein (GP) without the need for viral replication. However, the detailed molecular mechanisms underlying virus–host interactions remain largely unknown. Here, we show that EBOV-like particles (VLPs) formed by GP, VP40, and NP activate ECs in a GP-dependent manner, as demonstrated by the upregulation of intercellular adhesion molecules-1 (ICAM-1) expression. VLPs-mediated ECs activation showed a different kinetic pattern from that of TNF-α-mediated activation and was associated with apoptotic ECs disruption. In contrast to TNF-α, VLPs induced ICAM-1 overexpression at late time points. Furthermore, screening of host cytoskeletal signaling inhibitors revealed that focal adhesion kinase inhibitors were found to be potent inhibitors of ICAM-1 expression mediated by both TNF-α and VLPs. Our results suggest that EBOV GP stimulates ECs to induce endothelial activation and dysfunction with the involvement of host cytoskeletal signaling factors, which represent potential therapeutic targets for EVD.

## 1. Introduction

Ebola virus (EBOV), a member of the Filoviridae family, is an enveloped and negative-stranded RNA virus that causes severe hemorrhagic fever with high mortality rates in humans and other animals [1,2]. The genome of EBOV consists of seven genes encoding nucleoprotein (NP), RNA-dependent RNA polymerase (L), transmembrane glycoprotein (GP), matrix protein VP40, VP35, VP30, and VP24 [3,4]. Apart from the structural transmembrane GP, the transcription of the GP gene generates two nonstructural glycoproteins, the small soluble glycoprotein (ssGP) and the soluble glycoprotein (sGP), through transcriptional editing. The sGP, in turn, generates Δ-peptide as a result of its cleavage [5,6,7]. The cleavage of GP by the cellular metalloprotease TNFα-converting enzyme (TACE) generally occurs during EBOV infection, giving rise to shed GP, which structurally resembles the full-length GP and therefore can misdirect host-neutralizing antibodies directed against the full-length GP by presenting alternative epitopes [6,8,9].

GP can bind to multiple cell surface molecules, conferring on EBOV the ability to infect a wide range of cell types, including immune cells such as macrophages and dendritic cells, and other cells such as hepatocytes, epithelial cells, and endothelial cells (ECs) [10,11]. Vascular dysregulation leading to an increase in blood vessel permeability, loss of endothelial barrier integrity, and hemorrhage plays an important role in the severity of EBOV infection [1,12,13]. Despite their susceptibility to EBOV in vitro, ECs are considered late viral targets in vivo, and the molecular mechanisms underlying their impairment during EBOV infection remain elusive to date [8,9]. In addition to direct viral infection, the vascular endothelium can be targeted indirectly via mediators such as cytokines, which are released upon infection of primary target cells, including immune cells, as well as virus-encoded GPs, which in turn can target ECs either directly or through the activation of primary target cells [14,15,16,17]. Among the proposed working models, only GP and the soluble shed GP have been found to play a key role in the activation of ECs and a decrease in their barrier function. Neither viral infection and replication nor other virus-encoded GPs, such as sGP, Δ-peptide, and ssGP, were found to be necessarily involved in the activation of ECs [1,8,14,17,18]. As activation markers, the upregulation of cell adhesion molecules (CAMs), including intercellular adhesion molecules-1 (ICAM-1), was detected at the transcriptional level [14] and then at the protein level [1]. To confirm that EC activation depends on EBOV GP but does not require viral replication, Wahl-Jensen et al. [1] generated virus-like particles (VLPs) formed by VP40 and GP or only VP40 and demonstrated that only VLPs formed by VP40 and GP were able to activate human umbilical vein endothelial cells (HUVECs). However, whether the EBOV GP-mediated activation and disruption of ECs could be the result of its direct interaction with the cells via some receptors or an indirect effect by some cellular mediators induced by EBOV GP remains unclear.

Existing evidence suggests that overexpression of ICAM-1 on ECs can lead to increased vascular permeability and loss of the endothelial barrier, which is mediated by the activation of the Rho/ROCK pathway [19,20] and rearrangement of the actin cytoskeleton [21,22]. The activation of the Rho/ROCK pathway by ICAM-1 has been reported to initiate a positive feedback loop that could result in the expression of more ICAM-1 and the recruitment of more leukocytes [23] and that subsequently lead to diverse vasculopathies [24,25]. Junaid et al. [13] recently reported that the inhibition of the Rho/ROCK pathway by RevitaCell Supplement suppressed Ebola VLP-induced permeability increase in HUVECs, suggesting a possible involvement of this molecular pathway in EBOV disease (EVD) pathogenesis.

In the present study, we hypothesized that suppressing the Rho/ROCK pathway and some cytoskeletal signaling molecules would prevent the overexpression of ICAM-1 and associated ECs disruption observed during EBOV infection. First, we confirmed the upregulation of ICAM-1 expression in HUVECs after exposure to Ebola VLPs bearing GP as well as TNF-α, but not Ebola VLPs lacking GP. In contrast to TNF-α treatment, Ebola VLPs bearing full-length GP induced ICAM-1 overexpression at late time points. We also found that only Ebola VLPs treatment induced significant cytotoxicity, mainly apoptosis. Moreover, screening of the cytoskeletal signaling inhibitors library identified focal adhesion kinase (FAK) inhibitors as potent inhibitors of ICAM-1 mediated by both TNF-α and Ebola VLPs bearing GP. Our results suggest that EBOV GP stimulates ECs to induce endothelial activation and dysfunction with the involvement of host cytoskeletal signaling molecules, which represent potential therapeutic targets for EBOV infection.

## 2. Materials and Methods

### 2.1. Plasmids and Compound Library

Expression plasmids for glycoprotein (GP), nucleoprotein (NP), and viral matrix protein 40 (VP40) of Ebola virus (Mayinga strain, 1976 outbreak), used for the generation of Ebola virus-like particles (VLPs), were provided by Dr. Yoshihiro Kawaoka (The University of Tokyo, Japan) and Dr. Thomas Hoenen (Friedrich Loeffler Institute, Germany). A library of pharmacologically active cytoskeletal signaling-related compounds was purchased from Selleck Chemicals (Houston, TX, USA). This library contains United States Food and Drug Administration (FDA)-approved and registered drugs, as well as preclinical compounds.

### 2.2. Cell Culture

Human umbilical vein endothelial cells (HUVECs) (cat# D10013, Takara, Shiga, Japan,) were cultured in collagen-coated dishes (Corning, AZ, USA) containing endothelial growth medium (EGM) (Promocell, Heidelberg, Germany) supplemented with 10% fetal bovine serum (FBS), 1% penicillin/streptomycin solution, and 0.1% amphotericin B (Gibco, Grand Island, NY, USA). The human embryonic kidney cell line (HEK293T) (cat# CRL11268, ATCC, Manassas, VA, USA), used for the preparation of Ebola VLPs was maintained in Dulbecco’s modified Eagle’s medium (DMEM) supplemented with 10% FBS and 1% penicillin/streptomycin solution. All cells were incubated at 37 °C in a humidified 5% CO_2_ environment and were kept at a low passage number (below 20) for the experiments to maintain cell morphology and biological properties.

### 2.3. Ebola VLP Production and Purification

Ebola VLPs bearing GPs (designated VLPs) were generated as previously described [26], with some modifications. In brief, confluent HEK293T cells were cotransfected with pCAGGS-EBOV-GP, pCAGGS-EBOV-VP40, and pCAGGS-EBOV-NP using polyethyleneimine (PEI MAX) (Polysciences, Inc., Warrington, PA, USA). Following transfection, cells were incubated overnight, and the culture medium was replaced with fresh DMEM. Supernatants were collected after 48 h and clarified by centrifugation at 2500 rpm for 15 min at 4 °C. Ebola VLPs without GPs (designated VLP_VP40/NP_) were also generated by cotransfecting HEK293T cells with pCAGGS-EBOV-VP40 and pCAGGS-EBOV-NP. Transfection with pCAGGS alone served as a mock control. The collected supernatants containing Ebola VLPs and VLP_VP40/NP_ were layered onto 20% sucrose cushions and spun at 30,000 rpm at 4 °C for 2 h. The resultant pellets were resuspended in phosphate-buffered saline (PBS) and layered again onto 20–60% sucrose gradients for ultracentrifugation at 75,000 rpm for 2 h. The obtained fractions were harvested and analyzed by Western blotting, as described below. Further, EBOV protein-rich fractions were pooled and sedimented through a 20% sucrose cushion at 75,000 rpm for 30 min. The resulting pellet was resuspended in Opti-MEM serum-free medium (Thermo Fisher Scientific, Waltham, MA, USA), giving rise to purified Ebola VLPs and VLP_VP40/NP_ preparations. Purified Ebola VLPs and VLP_VP40/NP_ were quantified for GP content using Western blotting with recombinant GP lacking the transmembrane domain (EBOV rGPΔTM) (IBT Bioservices, Rockville, MD, USA) as the reference standard and anti-GP as the detection antibody. Unless otherwise stated, 10× diluted VLPs, corresponding to 9.2 µg/mL of GP, were used in most of the experiments, including drug screening.

### 2.4. Characterization of Ebola VLPs and Focal Adhesion Kinase Detection Using Western Blotting

Ebola VLPs and VLP_VP40/NP_ or the mock control particles were mixed with sodium dodecyl sulfate-containing buffer (Nacalai Tesque Inc., Kyoto, Japan), boiled for 5 min at 95 °C, and subjected to SDS-PAGE before being transferred onto a nitrocellulose blotting membrane (Amersham, Freiburg, Germany) overnight via Western blotting. The membranes were blocked in the blocking solution, made with the wash buffer (0.2 M Tris-HCl (pH 7.5), 8.76 g/L NaCl, and 0.25% Tween 20) containing 5% skim milk, for 1 h at 25 °C. After blocking, the membranes were first incubated overnight with mouse monoclonal anti-EBOV GP antibody provided by Dr. Ayato Takada (Hokkaido University, Japan) ([27]; 1:5000), and for 3 h with rabbit anti-VP40 serum ([28]; 1:5000) and rabbit polyclonal anti-EBOV NP antibody (IBT Bioservices; Rockville, MD, USA, 1:5000) separately. They were then probed with peroxidase-linked secondary antibodies, anti-mouse IgG-HRP (Merck, Darmstadt, Germany; 1:5000), or anti-rabbit IgG-HRP (Promega, Madison, WI, USA; 1:1000) for 1 h at room temperature. All antibodies were diluted in the blocking solution. The membranes were subsequently washed with the wash buffer three times for 10 min between primary and secondary antibody incubation, as well as after the secondary antibody incubation. Signals of bound secondary antibodies were detected using ECL prime chemiluminescent reagent (GE Healthcare, Chicago, IL, USA) and visualized using the LAS-3000 imaging system (Fujifilm, Tokyo, Japan). FAK was also detected in the cell lysates prepared from ECs treated with VLPs (10× dilution) and TNF-alpha (10 ng/mL) for 48 h, following the same protocol described above, using the rabbit monoclonal anti-FAK antibodies ([(D507U) XP, Cell signaling, Beverly, MA, USA; 1:1000; overnight]) and the corresponding secondary antibodies.

### 2.5. Treatment of Endothelial Cells (ECs) with Ebola VLPs or TNF-Alpha

ECs (2 × 10^4^) per well were plated in collagen-coated 96-well plates (Corning, NY, USA) for 24 h. The cells were then treated with 10× diluted Ebola VLPs and VLP_VP40/NP_ or, to study the dose-response effect, with 5×, 10×, 20×, 40×, 80×, and 160× diluted Ebola VLPs and VLP_VP40/NP_. As negative controls, ECs were either left untreated (mock) or treated with the mock control, which is the supernatant from the cells transfected with only pCAGGS. Cells treated with TNF-α at 10 ng/mL (Fujifilm Wako, Osaka, Japan) served as positive controls. Incubations were performed for up to 6, 12, 24, 48, and 72 h post-treatment at 37 °C in a humidified 5% CO_2_ environment. After incubation, the cells were fixed for immunofluorescence, as described below. In another set of experiments, confluent ECs were pretreated with RevitaCell Supplement (A2644501, Gibco, Grand Island, NY, USA, 1×) or increasing dilutions of focal adhesion kinase inhibitors from Selleck Chemicals (Houston, TX, USA) for 1 h before the addition of VLPs or TNF-α for 48 and 12 h incubation, respectively. The ECs were subsequently fixed for the evaluation of ICAM-1 expression through immunofluorescence assay.

### 2.6. Immunofluorescence Assay

For the evaluation of the expression level of ICAM-1 and CD31 (PECAM-1) in ECs, mouse monoclonal anti-ICAM-1 and anti-CD31 antibodies (MEM-111 and JC/70A, Abcam, Cambridge, UK) were used with 1000× dilution. After the appropriate treatment, the EC monolayers were fixed with 10% formalin overnight at 4 °C and then permeabilized with 0.2% Triton X-100 for 10 min at room temperature. Following permeabilization, the cells were blocked with 10% goat serum for 2 h at room temperature and incubated with specific primary anti-ICAM-1 and anti-CD31 antibodies overnight at 4 °C. A Corresponding secondary antibody conjugated to Alexa 488 (Thermo Fisher Scientific, Rockford, USA; 1:1000) was applied for 1 h at room temperature. Cells were washed with PBS four times for 5 min, and the resulting fluorescence was quantified with a SpectraMax iD5 microplate reader (Molecular Devices, San Jose, USA). Hoechst 33342 dye was added to stain cell nuclei, and cell images were obtained using a Cytation 5 imaging plate reader (BioTek Instruments, Winooski, VT, USA) with specific objectives. 

### 2.7. Apoptosis Detection

To detect apoptosis in cultured ECs following appropriate treatments, the CF488 terminal dUTP nick end labeling (TUNEL) apoptosis detection kit (Biotium, Fremont, CA, USA) was used according to the manufacturer’s instructions. Actinomycin D (Merck, Darmstadt, Germany) and ECs incubated with TUNEL reaction buffer without TdT enzyme were used as positive and negative controls, respectively. The fluorescence-positive cells representing apoptotic cells were quantified using the SpectraMax iD5 microplate reader, and images were acquired using a Cytation 5 imaging plate reader. 

### 2.8. Cell Viability Assay

Cytotoxic effects of VLPs on ECs were assessed using the Cell Counting Kit-8 (Gold Biotechnology, St. Louis, MO, USA). Cells were seeded at a density of 2 × 10^4^ cells per well into collagen-coated 96-well plates. After 24 h, the medium was removed, and the cultures were refed with 100 µL of medium alone as a negative control and with a medium containing either 10× diluted VLPs or to study the dose-response effect, VLPs at various concentrations. Non-seeded wells were treated similarly for blank measurements. The cytotoxic effect of TNF-α (10 ng/mL) was assessed following the same protocol. Forty-eight hours (or at a specific time point for the kinetic experiment) after the treatment, 10 µL of the Cell Counting Kit-8 reagent was added to each well, and the cultures were incubated at 37 °C for another 4 h before measuring the absorbance at 450 nm with a multi-mode microplate reader. The tetrazolium compound (WST-x8) contained in the assay reagent was reduced by live cells into a colored formazan that was measured at 450 nm, and its quantity was directly proportional to the number of live cells in the culture. In another set of experiments that were designed to confirm the cytotoxic effect of the focal adhesion kinase inhibitors, as well as the cytoskeletal signaling library compounds, the confluent ECs were pre-treated with 5 µM of library compounds and with serial dilutions of focal adhesion kinase inhibitors for 1 h before the addition of 10× diluted VLPs or TNF-α (10 ng/mL) for 48 and 12 h of incubation, respectively. Each experiment was performed in triplicates. Cell viability was calculated according to the manufacturer’s instructions and expressed as percentages. For the experiments using RevitaCell Supplement (A2644501, Gibco, Grand Island, NY, USA, 1×) to suppress the upregulation of VLPs- or TNF-α-mediated ICAM-1 expression, the viability of ECs was assessed at 48 and 12 h, respectively, as described above, following 1 h pretreatment with RevitaCell Supplement.

### 2.9. Drug Treatment Assays

For screening in 96-well plates, ECs were cultured for 24 h, as described above. Cultured media were removed, and cells were pretreated with the vehicle (DMSO or water) or the library compound for 1 h at 37 °C before the addition of TNF-α (10 ng/mL) or 10× diluted VLPs for 12 or 48 h post-treatment incubation, respectively. Cells were then fixed with 10% formalin and subjected to an immunofluorescence assay as described above. All compounds were used at a final concentration of 5 µM during the screening, and their inhibition of VLPs- or TNF-α-mediated ICAM-1 expression was determined by fluorescence measurement. Percent inhibition of ICAM-1 expression for each compound was determined by normalization to either VLPs- or TNF-α-treated wells without compounds. Following the same protocol, the experiment for the inhibition of micropinocytosis was performed with 30 min of incubation at 37 °C with 10 µM EIPA [5-(*N*-ethyl-*N*-isopropyl) amiloride] before the addition of 10× diluted VLPs or the medium (control) for 48 h.

### 2.10. Statistical Analysis

GraphPad Prism software, version 9.3.0. (463) (San Diego, CA, USA) was used for the determination of average values, standard errors, IC_50_, and CC_50_. Differences between groups were examined for statistical significance using either one-way or two-way ANOVA, with a *p* value < 0.05 considered as statistically significant. Relevant details of the analysis presented in individual figures are included in the accompanying figure legends. 

## 3. Results

### 3.1. Ebola VLPs Bearing GP Activate ECs and Induce Apoptosis

**Figure 1:**
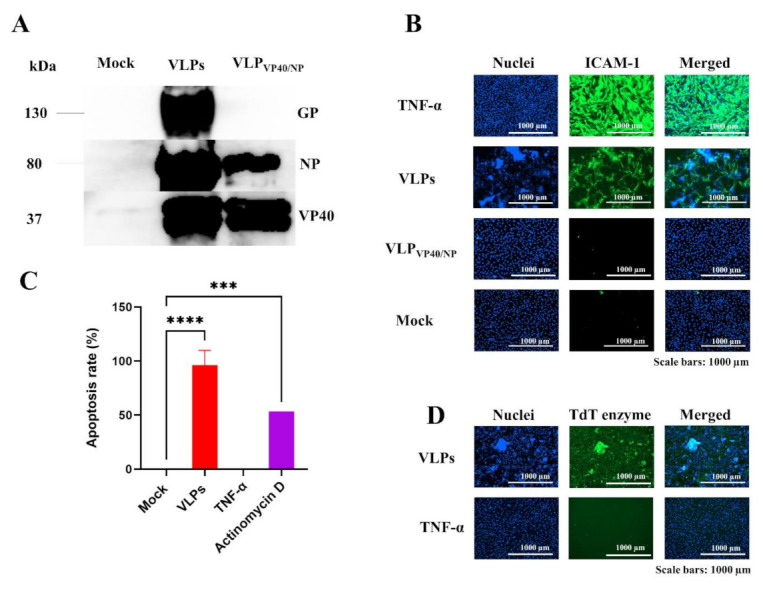

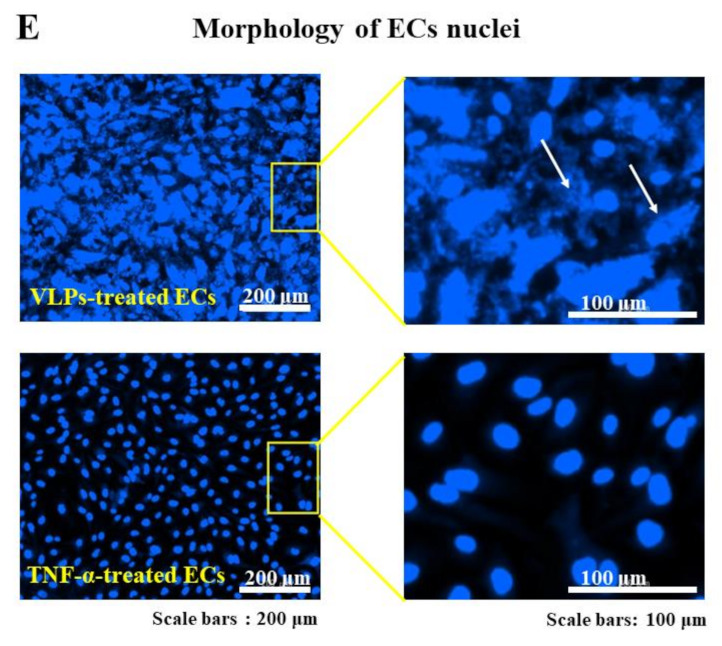
Ebola VLPs activate ECs and induce apoptosis. (**A**) The different VLPs preparations were purified from supernatants of transfected HEK293T cells and characterized using Western blotting with monoclonal antibodies (anti-GP, anti-VP40, and anti-NP). Supernatants from non-transfected cells were used as the negative control (Mock). Characterization with anti-GP indicated the absence of GP in VLP_VP40/NP_. (**B**) ECs were exposed to VLPs (10× dilution) or VLP_VP40/NP_ (10× dilution) for 48 h. Following the treatment, cells were fixed and permeabilized, and the activation was detected by immunofluorescence analysis using a monoclonal antibody directed against ICAM-1. The cells were then stained with Hoechst 33358 for imaging (magnification, ×10). Human recombinant TNF-α and supernatants from non-transfected cells (mock) were used as the positive and negative controls, respectively. Only cells treated with VLPs and TNF-α induced the expression of ICAM-1. Unlike TNF-α, VLPs-mediated upregulation of ICAM-1 expression was associated with cytopathic effects on ECs. Neither VLP_VP40/NP_ nor the mock control induced the upregulation of ICAM-1 expression. (**C**) The apoptotic rate was quantified using the fluorescence intensity of the nuclear staining with the TdT enzyme. Data represent the percent values of apoptotic cells derived from the normalization to the mock-treated and are expressed as mean ± SD (*n* = 2) from two independent experiments. *** *p* = 0.0006 and **** *p* < 0.0001 compared to the mock. The apoptotic rate in VLPs-, TNF-α- and Actinomycin D (4 µg/mL)-treated cells were compared to that in the cells treated with media (mock), applying one-way ANOVA, followed by Dunnett’s multiple comparisons test. (**D**) Images of nuclear staining from VLPs- and TNF-α-treated cells are shown, with VLPs-treated cells exhibiting apoptotic morphology in comparison to the TNF-α-treated cells. (**E**) Close up images showing the morphology of ECs nuclei after treatment with VLPs and TNF-α. White arrows indicate disrupted and aggregated nuclei in contact with VLPs. VLPs: virus-like particles; ECs: endothelial cells; ICAM-1: intercellular adhesion molecules 1.

In this study, we investigated and confirmed the upregulation of ICAM-1 expression on the surface of ECs following Ebola VLPs treatment. VLPs with GP (referred to as VLPs) and VLPs without GP (referred to as VLP_VP40/NP_) were generated by transient transfection of expression plasmids into HEK293T cells. Purified VLPs were detected by Western blotting, which was positive for the presence of viral structural proteins as determined with monoclonal antibodies (Figure 1A). ECs were treated with VLPs or VLP_VP40/NP_ for 48 h, and the activation of ECs was demonstrated by the upregulation of ICAM-1 expression using immunofluorescence assay. PECAM-1 expression level was also assessed to confirm the integrity of the EC monolayers (Figure S1). Similar to the positive control TNF-α (10 ng/mL), VLPs potently induced the upregulation of ICAM-1 expression on the surface of ECs (Figure 1B). VLPs exposure also led to the disruption of ECs with cells exhibiting typical apoptotic morphology with shrinkage and condensation of nuclei, as shown by nuclear staining. In contrast, no cytopathic effects were observed after the treatment of ECs with TNF-α. These results indicate that Ebola VLPs directly activate and disrupt ECs. Moreover, VLP_VP40/NP_ did not induce the upregulation of ICAM-1 expression or cytotoxicity (Figure 1B), suggesting that GP is required for vascular disruption.

Further, to confirm whether the observed cytopathic effects were due to apoptotic or necrotic cell death, fluorescent staining was performed using the TUNEL assay. In line with the results obtained with Hoechst staining, TUNEL staining showed a significant increase in the number of apoptotic cells among VLPs-treated ECs compared to that among TNF-α-treated ECs, similarly to the positive control actinomycin D treatment (Figure 1C). Images of nuclear staining confirmed the apoptotic morphology of cells treated with VLPs (Figure 1D), exhibiting condensed, aggregated, and disrupted nuclei (Figure 1E). Taken together, these results demonstrate that VLPs, but not TNF-α, induced ECs apoptosis in vitro.

### 3.2. ECs Disruption and ICAM-1 Expression Occur in a VLPs Dose-Dependent Manner

**Figure 2:**
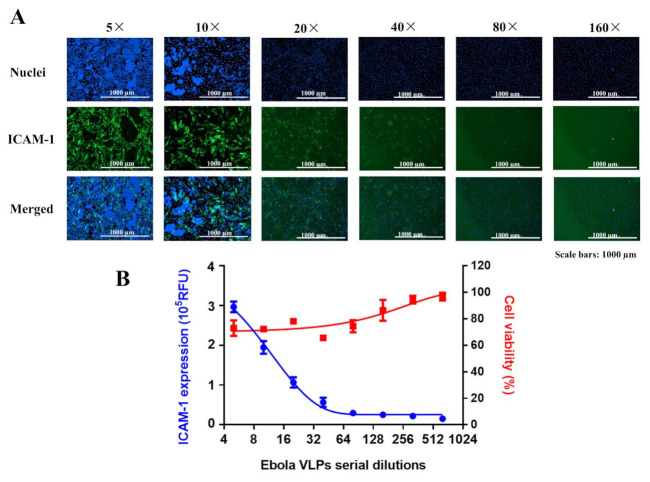
Ebola VLPs activate ECs in a dose-dependent manner. (**A**,**B**) ECs were treated with serial dilutions of VLPs for 48 h, fixed, and permeabilized for detection by immunofluorescence using an antibody directed against ICAM-1. Cell viability was determined using the Cell Counting Kit-8 assay. (**A**) Immunofluorescence images are shown for each VLPs dilution factor. (**B**) Dose-dependent curves showing induced ICAM-1 expression (blue) and cell viability (red). Data are representative of two independent experiments (*n* = 3). VLPs: virus-like particles; ECs: endothelial cells; ICAM-1: intercellular adhesion molecules 1.

To determine whether VLPs cause endothelial disruption in a dose-dependent manner, ECs were treated with different concentrations of VLPs. Our results showed that ICAM-1 expression and cell disruption levels increased in a dose-dependent manner with VLPs (Figure 2A). The cytotoxic effect of VLPs was also confirmed by the cell counting kit-8 cytotoxicity assay, which is a colorimetric assay for the determination of cell viability. The high intensity of ICAM-1 expression with significant damage to ECs (shown with the reduction of cell viability) was observed with 5× and 10× diluted VLPs (Figure 2A,B). The amount of GP contained in 10× diluted VLPs, which was used in all downstream experiments, was estimated using serial dilutions of recombinant GP without the transmembrane region (EBOV rGPΔTM) by Western blot analysis and quantification. A linear curve was obtained, and 10× diluted VLPs samples were found to contain 9.2 g/mL GP (Figure S2). Further, we demonstrated that the level of ECs injury was proportional to the level of ICAM-1 expression, which was low or almost inexistent in the presence of 40×, 80×, and 160× diluted VLPs, with no observed ECs disruption or reduction in the number of viable cells.

### 3.3. VLPs- and TNF-α-Induced ICAM-1 Upregulation Follow Different Kinetic Patterns

**Figure 3:**
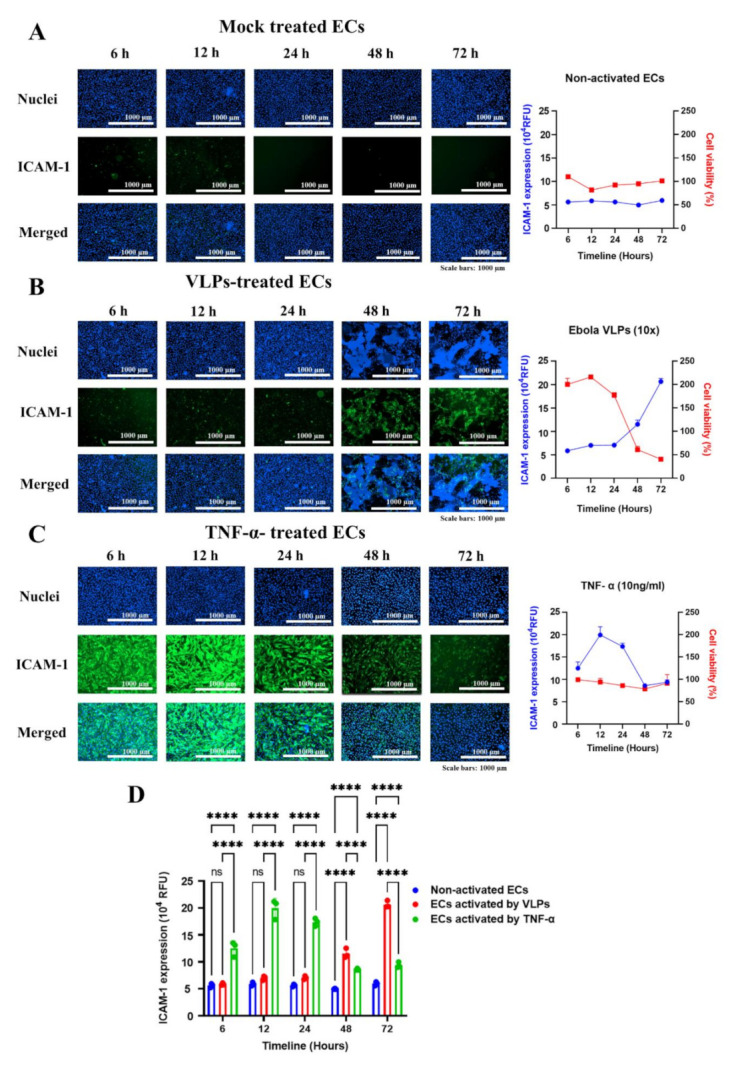
Kinetics of ICAM-1 protein expression. (**A**–**C**) ECs were exposed to VLPs (10× dilution), TNF-α (10 ng/mL), or cell medium only (mock) for 6, 12, 24, 48, and 72 h. Following the treatment at each designated time point, cells were fixed and permeabilized for the detection of ICAM-1 protein expression by immunofluorescence using antibodies directed against ICAM-1. Related cell viability at each time point was evaluated using the CCK-8 assay. (**A**) No detection of ICAM-1 expression (left) and no change in viability was observed in non-activated ECs (right). (**B**) A significant increase in ICAM-1 protein expression levels was shown with VLPs treatment from 48 h post-treatment. The cell viability was inversely proportional to the ICAM-1 protein expression and was reduced drastically from 48 h post-treatment. (**C**) TNF-α-treated cells presented an early signal of ICAM-1, with maximal levels of expression at 12 h post-treatment and a further decrease over time. (**D**) The summary graph of the kinetics of ICAM-1 protein expression. The results are presented as averages, and error bars indicate standard deviation (*n* = 3). Statistical significance is shown: **** *p* < 0.0001. VLPs and TNF-α-treated cells were compared, and both were also compared with the non-activated cells applying two-way ANOVA and followed by Tukey’s multiple comparisons test. ICAM-1: intercellular adhesion molecules 1; ECs: endothelial cells; VLPs: virus-like particles.

To examine the kinetics of ICAM-1 induction on the surface of ECs, cells were treated with VLPs, TNF-α (10 ng/mL), or medium alone (mock-treated), and the resulting expression of ICAM-1 was detected at 6, 12, 24, 48, and 72 h after treatment. Additionally, we investigated the kinetics of ECs viability following treatment using the cell counting kit-8 assay. ICAM-1 expression was not detected on ECs treated with medium alone, and there was no change in their viability over time (Figure 3A). In contrast, VLPs-treated cells showed a significant increase in ICAM-1 expression from 48 h post-treatment, with cells presenting cytopathic effects (Figure 3B). Compared to ECs treated with TNF-α (Figure 3C), ECs treated with VLPs showed a drastic increase in cell viability from 6 h with a peak at 12 h before decreasing from 48 h (Figure 3B, graph to the right). Early expression of ICAM-1 by TNF-α treatment was detected at 6 h post-treatment and increased significantly at 12 h post-treatment before decreasing over time (Figure 3C). Cell viability under TNF-α treatment was not significantly affected, and no cytopathic effects were observed (Figure 3C). Overall, ICAM-1 expression was observed early with TNF-α treatment compared to the late expression with VLPs treatment (Figure 3D), associated with cell disruption. These results suggest that ECs activation and disruption induced by VLPs and TNF-α follow different kinetic patterns.

**Figure 4:**
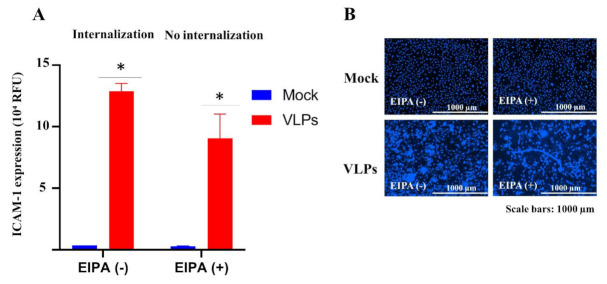
Micropinocytosis inhibition did not suppress VLPs-induced ICAM-1 upregulation and associated cytopathic effects. ECs were pretreated with 10 µM EIPA for 30 min at 37 °C before the addition of VLPs. The cells were incubated for 48 h at 37 °C, and the expression level of ICAM-1 on ECs and the morphology of ECs were assessed using immunofluorescence and nuclei staining, respectively. ECs treated with the medium (mock) were used as control. (**A**) Graphic representation of ICAM-1 expression. (**B**) Images of stained nuclei showing the cytopathic effect following the exposure to VLPs. Data are the representative of two experiments performed in duplicate and are presented as the mean ± SD. Statistical significance is shown: * *p* ≤ 0.05. VLP-treated ECs with EIPA and without EIPA were compared applying two-way ANOVA and followed by Šídák’s multiple comparisons test. VLPs: virus-like particles; ECs: endothelial cells; ICAM-1: intercellular adhesion molecules 1.

To further understand the difference in the kinetics of ICAM-1 expression between VLPs and TNF-α we questioned whether VLPs-mediated ICAM-1 expression upregulation is the result of a stimulation from the cell surface attachment or a cytokine response following the internalization. ECs were pretreated with the medium or EIPA [5-(*N*-ethyl-*N*-isopropyl) amiloride], an inhibitor of the Na+/H+ exchanger that specifically inhibits micropinocytosis [29,30], for 30 min at 37 °C, before the addition of VLPs or the medium (control) for 48 h. Pretreatment of ECs with EIPA did not prevent the VLPs-mediated ICAM-1 expression upregulation (Figure 4A) and the associated cytopathic effects (Figure 4B) to occur. These results suggest that the induction of ICAM-1 expression upregulation and the cytopathic effect might be the result of the stimulation following the cell surface attachment. VLPs do not need to be internalized to induce ICAM-1 upregulation and cytopathic effects on ECs. 

### 3.4. Inhibition of the Rho/ROCK Pathway Reduced VLPs- and TNF-α-Induced ICAM-1 Upregulation

**Figure 5:**
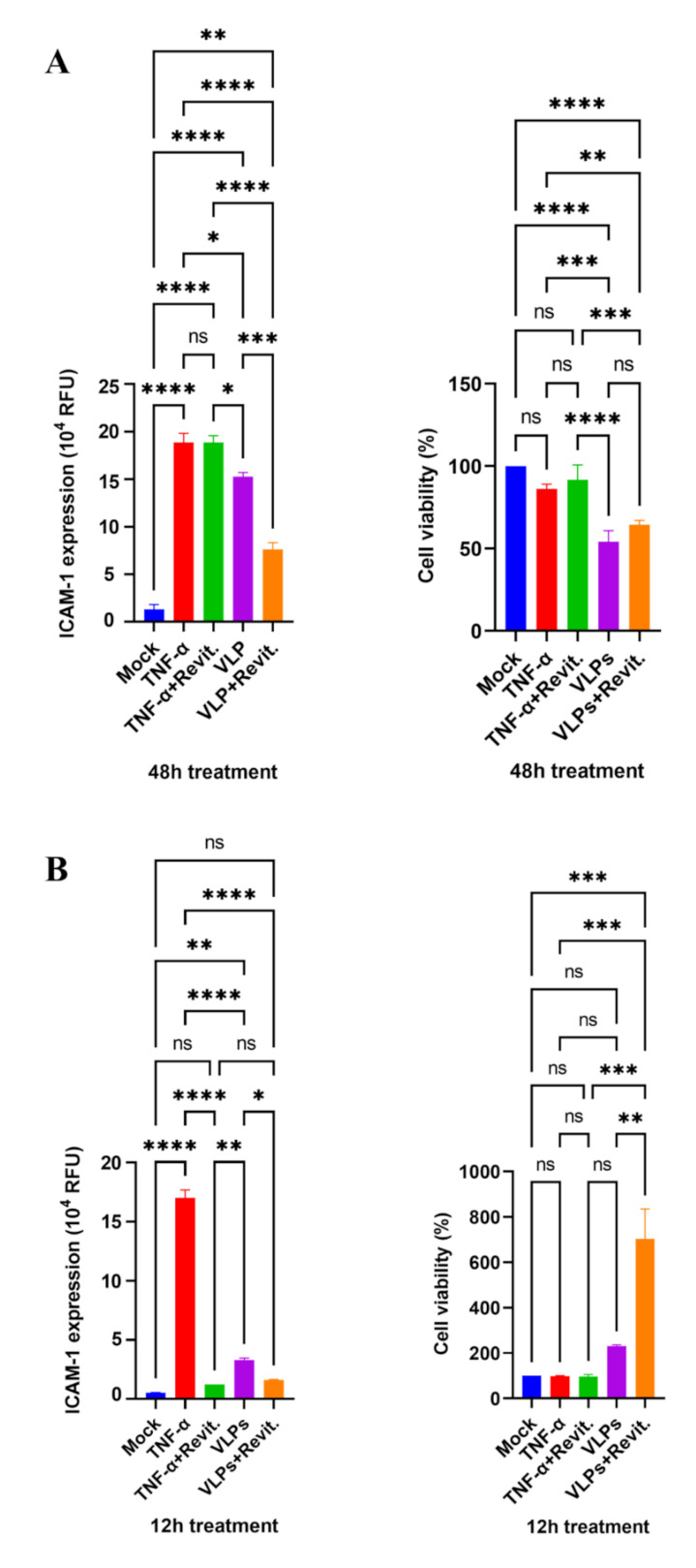
Effect of RevitaCell on VLPs- and TNF-α-induced ICAM-1 upregulation. (**A**) ECs were pre-treated for 1 h with RevitaCell (1×) and then treated with VLPs (10×) or TNF-α (10 ng/mL). Forty-eight hours after treatment, cells were fixed and immunostained for fluorescence measurement. For each condition, the percentage of ICAM-1 expression was calculated as the ratio of the expression level in the wells pre-treated with RevitaCell to the expression level in wells treated with either VLPs or TNF-α only. Cell viability was also assessed using the cell counting kit-8 according to the manufacturer’s recommendations. Data for ICAM-1 expression and cell viability in the presence of RevitaCell are shown, representing mean ± SD of triplicate results from two independent experiments. (**B**) To study the effect of RevitaCell at early incubation time, a similar pre-treatment with RevitaCell (1×) was performed. Fluorescence was measured 12 h after the addition of VLPs (10×) or TNF-α (10 ng/mL). EC viability was similarly assessed 12 h after appropriate treatments. Data show relative VLPs- or TNF-α-mediated ICAM-1 expression levels with or without RevitaCell (left), as well as cell viability under corresponding treatment (right). One-way ANOVA followed by Tukey post-test was applied to compare data, which are presented as mean ± SD of triplicate results. Statistical significance is shown: **** Extremely significant *(p* < 0.0001), *** Extremely significant (*p* = 0.0001 to 0.001), ** Very significant (*p* = 0.001 to 0.01), * Significant (*p* = 0.01 to 0.05), ns not significant (*p* ≥ 0.05). VLPs: virus-like particles; ECs: endothelial cells; ICAM-1: intercellular adhesion molecules 1.

The induction of ICAM-1 expression on ECs mediates the activation of the Rho/ROCK pathway, which modulates vascular permeability [24,25]. Inhibition of the Rho/ROCK pathway by RevitaCell Supplement has been recently reported to suppress EBOV-induced permeability increase in ECs [13]. Therefore, we tested the effect of Rho/ROCK pathway inhibition on VLPs-mediated ECs activation. ECs were pretreated with RevitaCell Supplement for 1 h at 37 °C before the addition of either VLPs or TNF-α. First, ICAM-1 expression and cell viability were assessed after 48 h of incubation. Results showed that VLPs-induced ICAM-1 expression level decreased upon RevitaCell Supplement administration, while TNF-α-induced ICAM-1 expression level was not affected (Figure 5A, left). The administration of RevitaCell Supplement in the presence of VLPs and TNF-α did not affect EC viability at this time point (Figure 5A, right). Further, following TNF-α-induced ICAM-1 expression at early time points, we investigated whether RevitaCell Supplement affects TNF-α-induced ICAM-1 expression and cell viability at 12 h after treatment, which was the peak time point of ICAM-1 expression induced by TNF-α. As shown in Figure 5B, TNF-α-induced ICAM-1 expression was almost completely inhibited at this time point. The slight induction of ICAM-1 expression by VLPs was also suppressed by RevitaCell Supplement treatment. However, while the viability of ECs treated with TNF-α was not affected by the addition of RevitaCell Supplement, we observed more than a two-fold increase in survival rate following VLPs treatment and approximately a seven-fold increase upon the addition of RevitaCell Supplement (Figure 5B, right), similar to what we observed in Figure 3B. 

Taken together, these results suggest that TNF-α and VLPs induce ICAM-1 expression in different kinetic modes involving the Rho/ROCK pathway. The modulation of the Rho/ROCK pathway differs and has different effects on ECs depending on the stimulator, which could be TNF-α or VLPs. Moreover, Ebola VLPs induced cell proliferation at early time points, which was enhanced by the inhibition of the Rho/ROCK pathway.

### 3.5. Screening of Host Cytoskeletal Signaling Inhibitors Identified Compounds Inhibiting VLPs-Mediated ECs Activation

**Figure 6:**
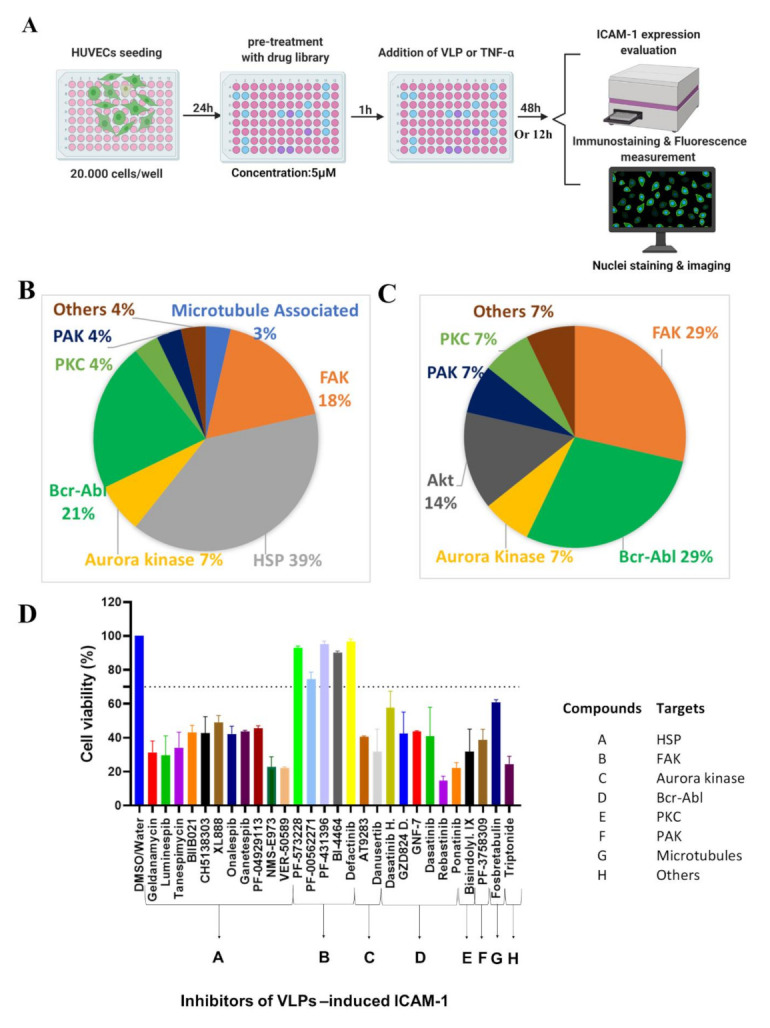

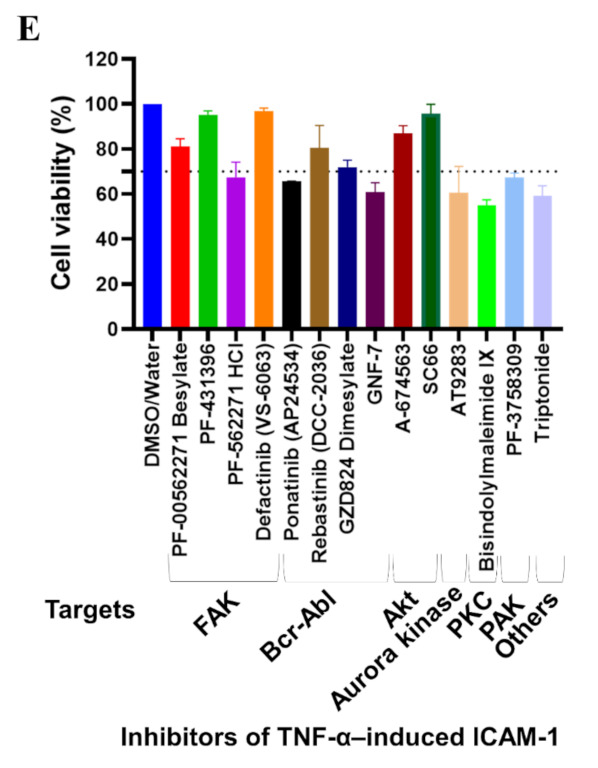
Screening of host cytoskeletal signaling compounds inhibiting VLPs-mediated endothelial cells activation (**A**) Illustration of the screening strategy. The screening was conducted on VLPs- or TNF-α-treated cells. Compounds (*n* =179) were evaluated for their effects on the inhibition of ICAM-1 expression. The fluorescence corresponding to the expression of ICAM-1 was measured 12 and 48 h post-treatment for TNF-α- or VLPs-treated cells, respectively, following immunostaining. The protective effects of the compounds were assessed through nuclear staining and imaging. RevitaCell Supplement was used as the positive control. Compounds exhibiting 70% inhibition of ICAM-1 expression were considered effective inhibitors, with 28 and 14 of them inhibiting VLPs- and TNF-α-induced ICAM-1 expression, respectively. (**B**) The target host proteins for VLPs-induced ICAM-1 inhibitors and (**C**) TNF-α-induced ICAM-1 inhibitors are shown. (**D**) The inhibitors of VLPs-induced ICAM-1 expression targeting FAK and (**E**) those of TNF-α-induced ICAM-1 expression targeting FAK, Akt, and Bcr-Abl showed less than 30% cytotoxicity (limit shown by grid lines at 70% cell viability). VLPs: virus-like particles; ECs: endothelial cells; ICAM-1: intercellular adhesion molecules 1; Akt: Ak strain transforming kinase; FAK: Focal adhesion kinase; Bcr-Abl: breakpoint cluster region-abelson.

The Rho/ROCK pathway is an important mediator of host cytoskeletal signaling [31,32,33]. Because the inhibition of the Rho/ROCK pathway by RevitaCell Supplement reduced the expression of ICAM-1 induced by both Ebola VLPs and TNF-α, we hypothesized that screening a library of cytoskeletal signaling inhibitors, which consists of known targets, represents an effective approach to identify therapeutic targets for protecting ECs from virus-associated damage. Thus, 179 compounds were evaluated for their effects on the inhibition of ICAM-1 expression in VLPs- or TNF-α-treated ECs. An initial screening was performed by treating ECs with each compound at a concentration of 5 µM in the presence of either VLPs or TNF-α. The expression of ICAM-1 was assessed at 48 and 12 h after treatment with VLPs and TNF-α, respectively, and the protective effect of each compound was evaluated by nuclear staining and imaging (Figure 6A).

Upon completion of the initial screening (Table S1), 28 and 14 compounds inhibiting VLPs- and TNF-α-induced ICAM-1 expression were respectively identified at >70% inhibition level. The corresponding targets of VLPs- and TNF-α-induced ICAM-1 inhibitors are shown in Figure 6B, C, respectively. Importantly, we noticed that most of the inhibitors targeted two host proteins in both VLPs- and TNF-α-mediated ICAM-1 expression: the breakpoint cluster region-abelson (Bcr-Abl) protein and focal adhesion kinase (FAK) (Figure 6B,C). Heat shock proteins (HSP) were identified as the highest hit targets specific to VLPs-induced ICAM-1 expression. None of the inhibitors of TNF-α-induced ICAM-1 expression targeted the heat shock proteins.

To exclude the possibility that the inhibitory effect of the compounds is related to their cytotoxicity, we evaluated the cytotoxicity of identified compounds on ECs using the cell counting kit-8 assay. When compounds with <30% cytotoxicity were selected, only the inhibitors of FAK were found to meet the cytotoxicity criteria in the VLPs-induced ICAM-1 expression inhibitor group (Figure 6D). In the TNF-α-induced ICAM-1 inhibitor group, eight potent compounds targeting Bcr-Abl, Ak strain transforming (Akt) kinase, and FAK were found to be less toxic (Figure 6E). Therefore, FAK inhibitors were selected and further investigated. The inhibitors of HSP, which were potent inhibitors and specific to VLPs, showed significant cytotoxicity in ECs, suggesting their non-specific inhibitory effect (Figure 6D).

### 3.6. FAK Inhibitors Block VLPs- and TNF-α-Induced ECs Activation

**Figure 7:**
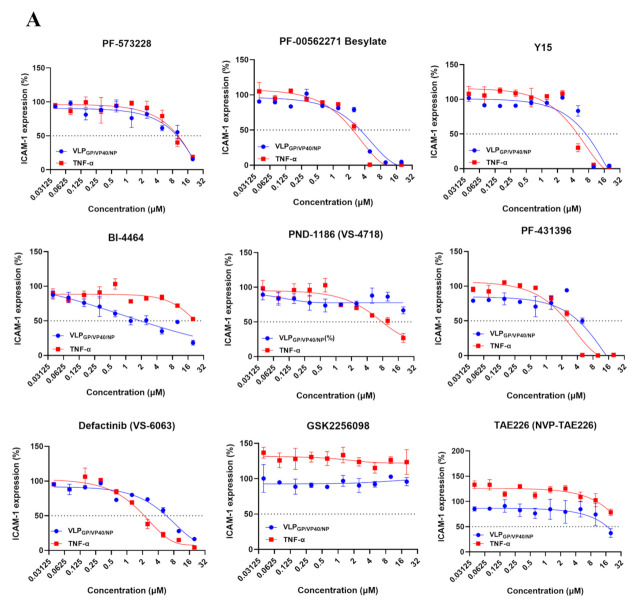

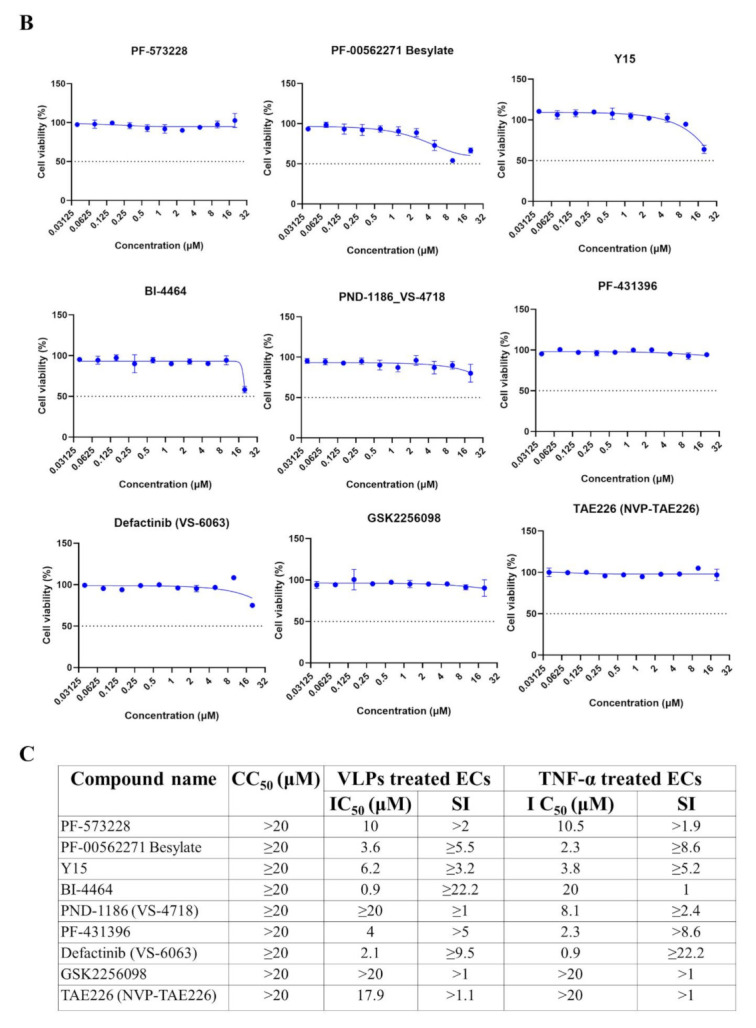
Dose-response curves of potent FAK inhibitors. (**A**) ECs were pretreated for 1 h at 37 °C with 10 increasing doses (0.0390625, 0.078125, 0.1562, 0.3125, 0.625, 1.25, 2.5, 5, 10, and 20 µM) of PF-573228, PF-00562271 Besylate, Y15, BI-4464, PND-1186 (VS-4718), PF-431396, Defactinib (VS-6063), GSK2256098, and TAE226 (NVP-TAE226) before the addition of TNF-α (10 ng/mL) or VLPs (10×) for 12 and 48 h incubation, respectively. Cells were then subjected to immunofluorescence assay for ICAM-1 evaluation. ICAM-1 inhibition percentage of each compound corresponds to the relative ICAM-1 expression induced by VLPs or TNF-α in the presence of the compound, which was normalized to the relative ICAM-1 expression induced by VLPs or TNF-α only. In another set of experiments, (**B**) ECs were treated for 48 h with the same concentration range of the above-mentioned compounds and subjected to cytotoxicity analysis. Data represent the mean ±SD (error bars) of triplicate results from two independent experiments. (**C**) The IC_50_ and CC_50_ values for each compound are shown with their corresponding SI (CC_50_/IC_50_). FAK: Focal adhesion kinase; ECs: endothelial cells; VLPs: virus-like particles; ICAM-1: intercellular adhesion molecules 1.

To assess the potency and specificity of the selected FAK inhibitors on VLPs- or TNF-α-treated cells, we tested their dose-dependency at concentrations up to 20 µM. The cytotoxicity of the compounds was also evaluated in the same concentration range, including all nine FAK inhibitors contained in the screened library. Among them, seven and eight compounds dose-dependently inhibited VLPs- and TNF-α-induced ICAM-1 expression, respectively. As shown in Figure 7A, in the VLPs-treated ECs, four compounds showed high potency with IC_50_ < 5 µM. BI-4464 was the most potent with an IC_50_ of 0.9 µM, followed by Defactinib (VS-6063) with an IC_50_ of 2.1 µM, PF-00562271 Besylate with an IC_50_ of 3.6 µM, and PF-431396 with an IC_50_ of 4 µM. Y15, PF-573228, and TAE226 (NVP-TAE226) were also effective with IC_50_ values > 5 µM and displayed low toxicity in ECs. Additionally, BI-4464 demonstrated a selective index (SI) of 22.2, indicating its highly specific inhibitory effect (Figure 7C). Four potent compounds showed IC_50_ < 5 µM in the TNF-α-treated group, including Defactinib (VS-6063) with the highest IC_50_ value (0.9 µM), PF-00562271 Besylate with an IC_50_ of 2.3 µM, PF-431396 with an IC_50_ of 2.3 µM, and Y15 with an IC_50_ of 3.8 µM (Figure 7A,C). GSK2256098 affected neither VLPs- nor TNF-α-induced ICAM-1 expression. Taken together, Defactinib (VS-6063) could be considered a potent inhibitor of both VLPs- and TNF-α-induced ICAM-1 expression, with SI ranging between 9.5 and 22.2. BI-4464, which showed potency in VLPs-treated ECs, did not potently inhibit ICAM-1 expression in TNF-α-treated ECs. As shown in Figure 7B, none of the compounds showed toxicity on untreated ECs (CC_50_ ≥ 20 µM).

In both cases, the inhibition of ICAM-1 expression had no effect on ECs viability. Contrary to our hypothesis, the images of ECs nuclei revealed that the selected compounds could suppress the expression of ICAM-1 mediated by VLPs and TNF-α in a dose-dependent manner without rescuing the cells from VLPs-induced injury (Figure S3).

Since FAK inhibitors reduced both VLPs- and TNF-α-induced ICAM-1 expression in ECs, we checked, by Western blot analysis, whether FAK protein expression was increased upon treatment with VLPs or TNF-α. The results in Figure S4 showed that VLPs and TNF-α did not increase the basal level of FAK expression. Therefore, the consistent expression of FAK may have simply contributed to the activation by VLPs and TNF-α, which may have led to the upregulation of ICAM-1 expression in ECs.

## 4. Discussion

Dysfunction and injury of ECs constitute the major outcomes of EBOV infection and play key roles in disease development. Previous studies have indicated that EBOV GP is the main determinant of EC activation and cytotoxicity [1,17]. However, the mechanisms underlying the interactions between EBOV GP and ECs are largely unknown. In this study, we found that, similar to TNF-α, Ebola VLPs bearing GP (referred to as VLPs) could activate ECs, as confirmed by the upregulation of ICAM-1 expression on the cell surface (Figure 1B). In addition to the induction of ICAM-1 expression, the exposure of ECs to VLPs led to the appearance of cytopathic effects (Figure 1B), which were due to apoptosis, while TNF-α activation of ECs was not associated with apoptosis and subsequent cytopathic effects (Figure 1C–E). In contrast to VLPs, GP-deficient VLPs (referred to as VLP_VP40/NP_) did not activate ECs, as shown by the absence of ICAM-1 expression (Figure 1B), suggesting that GP is required for vascular activation and disruption. These results are consistent with those of previous studies indicating that GP is the main determinant of vascular injury and cytotoxicity and that viral replication is not required for the vascular dysfunction observed during EBOV infection. 

The induction of ICAM-1 expression on the surface of ECs and the associated apoptotic cell death occurred in a VLPs dose-dependent manner (Figure 2A), and the level of the observed cytotoxicity was found to be directly dependent on the level of EBOV GP. Previously, it has been demonstrated that the cytotoxicity of EBOV is significantly increased by the overexpression of GP [34], which has been further supported by Ray et al. [35], who reported that the expression of EBOV GP in recombinant adenovirus (Ad-Ebola GP)-transduced primary human cardiac microvascular endothelial cells and HUVECs induced apoptotic cell death with related cytopathic effects in a dose-dependent manner, demonstrating the importance of GP expression level in the cytotoxic effect through apoptosis induction. 

In agreement with these findings, we showed that ECs treated with 10× diluted VLPs, which contained approximately 9.2 µg/mL of GP, exhibited a strong cytopathic effect than that of ECs treated with 160× diluted VLPs, which contained a low amount of GP (Figure 2). 

The relevance of EBOV GP level on the induction of ECs apoptosis and disruption suggests a direct correlation between disease severity and the presence of a high level of shed GP and high viral load in the serum of EVD patients, as reported elsewhere [36]. 

ICAM-1 expression is constitutively low at the surface of ECs and may be upregulated in response to stimuli, such as viral infection, oxidative stress, and pro-inflammatory cytokines, including interleukin-1β (IL1β), TNF-α, and interferon-γ (IFN-γ) [37,38]. Kinetic studies of ICAM-1 expression upon activation with TNF-α or VLPs revealed a difference in kinetics patterns. TNF-α-mediated upregulation of ICAM-1 occurred early around 6 h post-treatment and reached a peak at 12 h before decreasing around 48 h, with no cytopathic effect observed in ECs over time (Figure 3C). Previous studies have shown that TNF-α can activate the endothelium and modulate its barrier function in tissue-culture models and in vivo while maintaining a relatively intact morphology [2]. This observation was confirmed in our study, where the activation of the endothelium by TNF-α was not associated with a direct induction of ECs disruption and the appearance of cytopathic effects. In contrast, VLP-mediated upregulation of ICAM-1 appeared at a later time point (48 h post-treatment), with cells presenting injury and typical apoptotic morphology. A correlation between the levels of ECs disruption and the expression of ICAM-1 was observed (Figure 3B). While the treatment of ECs with TNF-α did not affect cell viability and growth, ECs exposure to VLPs increased the survival rate of the cells by approximately 200% from 6 h, with a peak at 12 h before decreasing from 48 h (Figure 3B, graph at the right). To the best of our knowledge, our study is the first to show that Ebola VLPs upregulate host cell proliferation within several hours following treatment before inducing apoptosis. We speculate that the EBOV VLP-induced abnormal cell proliferation might be the result of the cell stress response, which in turn has triggered the subsequent apoptosis after failing to cope with the insult caused by VLPs [39]. More studies to understand the interplay between the GP on the VLPs, the ECs proliferation, and the apoptosis induction are needed. 

The level of ICAM-1 expression in VLP-treated ECs was not upregulated before 48 h of incubation. Connolly-Andersen et al. observed the same kinetic pattern for ICAM-1 upregulation at the transcriptional level following the activation of HUVECs with various doses of Crimean-Congo hemorrhagic fever virus (CCHFV) [38]. Wahl-Jensen et al. reported the upregulation of ICAM-1 at 12 and 24 h at the level of gene transcription after treatment of HUVECs with Ebola VLPs consisting of GP and VP40 and at the protein level after the infection of HUVECs with live EBOV [1]. Whether the expression observed at the cell surface was a basal ICAM-1 expression, as no cytopathic effect was reported together with the ICAM-1 upregulation in HUVECs, needs to be clarified. One of the limitations of our assay was the evaluation of ECs activation only with the induction of ICAM-1 upregulation, which cannot provide more details such as ECs contraction and ultrastructural changes. Transmission electron microscopy of ECs undergoing apoptosis in the presence of the VLPs in comparison with the mock-treated ECs would provide a deep understanding of the VLPs’ effect on ECs. 

The involvement of the Rho/ROCK pathway in the induction of hyperpermeability in HUVECs treated with Ebola VLPs [13] led us to hypothesize that the inhibition of the Rho/ROCK pathway would suppress TNF-α- and VLP-induced ICAM-1 upregulation, as well as VLP-induced apoptotic disruption in ECs. Our hypothesis was verified for only the suppression of ICAM-1 expression (Figure 5A,B). The kinetic patterns of TNF-α and VLP activities had an impact on the modulation of the Rho/ROCK pathway, as seen by RevitaCell suppression of TNF-α-induced ICAM-1 at 12 h (Figure 5B) and VLP-induced ICAM-1 at 48 h (Figure 5A). Several studies have reported that the inhibition of Rho/ROCK does not reverse the increase in EC permeability for several hours after TNF-α exposure and stimulation [40,41,42]. Consistent with these reports, the inhibition of the Rho/ROCK pathway in our study did not influence TNF-α-induced ICAM-1 expression upregulation at 48 h after treatment. Furthermore, the inhibition of the Rho/ROCK pathway led to the abnormal cell proliferation of ECs exposed to VLPs at 12 h of incubation, suggesting a probable interaction with downstream effectors or pathways that might be specific to the VLPs and responsible for the observed apoptosis. 

Since the Rho/ROCK pathway controls EC dysfunction through the regulation of cytoskeleton organization [23], we screened the inhibitors of host cytoskeleton signaling pathways and identified FAK inhibitors as potent inhibitors of ICAM-1 upregulation mediated by both TNF-α and VLPs (Figure 6B,C). Among the nine FAK inhibitors analyzed for their dose-response activity, BI-4464 was the most effective against VLPs-induced ICAM-1 expression, while Defactinib (VS-6063) showed potency against both VLPs- and TNF-α-induced ICAM-1 expression (Figure 7).

Unexpectedly, FAK inhibitors did not prevent Ebola VLPs-induced apoptosis in ECs, suggesting the possible existence of an additional host signaling pathway, acting either independently of FAK, or at triggering GP-mediated apoptosis at the upstream of FAK pathway associated with ICAM-1 induction. Further investigation of other identified compounds targets, including HSP and Bcr-Abl, may be key to unraveling the molecular mechanisms of GP-induced apoptosis. To the best of our knowledge, this is the first study identifying FAK as a therapeutic target for EVD pathogenesis. FAK is overexpressed in various cancers and has been found to play key roles in normal and cancer cellular functions, such as survival and proliferation [43]. Notably, it has been reported as an anticancer target. As some of our identified compounds are in clinical test phases, the FAK inhibitors constitute potential therapeutic drugs or lead compounds for further development, which target disease pathogenesis related to endothelial barrier dysfunction in severe cases of EVD. 

## 5. Conclusions

In summary, this study showed that EBOV-like particles (VLPs)-bearing GP could induce ECs apoptotic disruption and activation, as shown by intercellular adhesion molecules-1 (ICAM-1) upregulation, without requiring internalization into the cell. Additionally, we found that the inhibitors of certain cytoskeletal signaling molecules such as focal adhesion kinase could inhibit both VLPs and TNF-α-induced upregulation of ICAM-1 expression without rescuing ECs from the GP-induced apoptotic disruption. The results suggest that Ebola GP triggers apoptosis through an additional pathway other than the ICAM-1 induction pathways. Finally, the cytoskeletal signaling pathways may serve as important targets for the development of therapeutic drugs against EBOV disease. 

## Data Availability

All available data are presented in the article.

## Supplementary Materials

The following are available online at https://www.mdpi.com/article/10.3390/v14010142/s1, Figure S1: Platelet endothelial cell adhesion molecule 1 (PECAM-1) staining to confirm the integrity of the endothelial monolayer; Figure S2: Quantification of Ebola GP contained in the produced Ebola Virus-like particles (VLPs); Figure S3: Nuclei staining of virus-like particles (VLPs)-treated endothelial cells in the presence of focal adhesion kinase (FAK) inhibitors; Figure S4; FAK detection in endothelial cells.; Table S1: Summary of cytoskeletal library screening for the inhibition of virus-like particles (VLPs)- and TNF-α-induced intercellular adhesion molecules 1 (ICAM-1) expression.
