# Supplementary material for: Ebola Virus GP Activates Endothelial Cells via Host Cytoskeletal Signaling Factors
Source: Viruses. 2022 Jan 13;14(1):142. doi: 10.3390/v14010142 (PMC8781776; doi:10.3390/v14010142)
Supplement: Supplementary file 1 [file viruses-14-00142-s001.zip › viruses-1552458-supplementary.pdf]

viruses

Supplementary Materials

Ebola Virus GP Activates Endothelial Cells via Host Cytoskeletal Signaling Factors

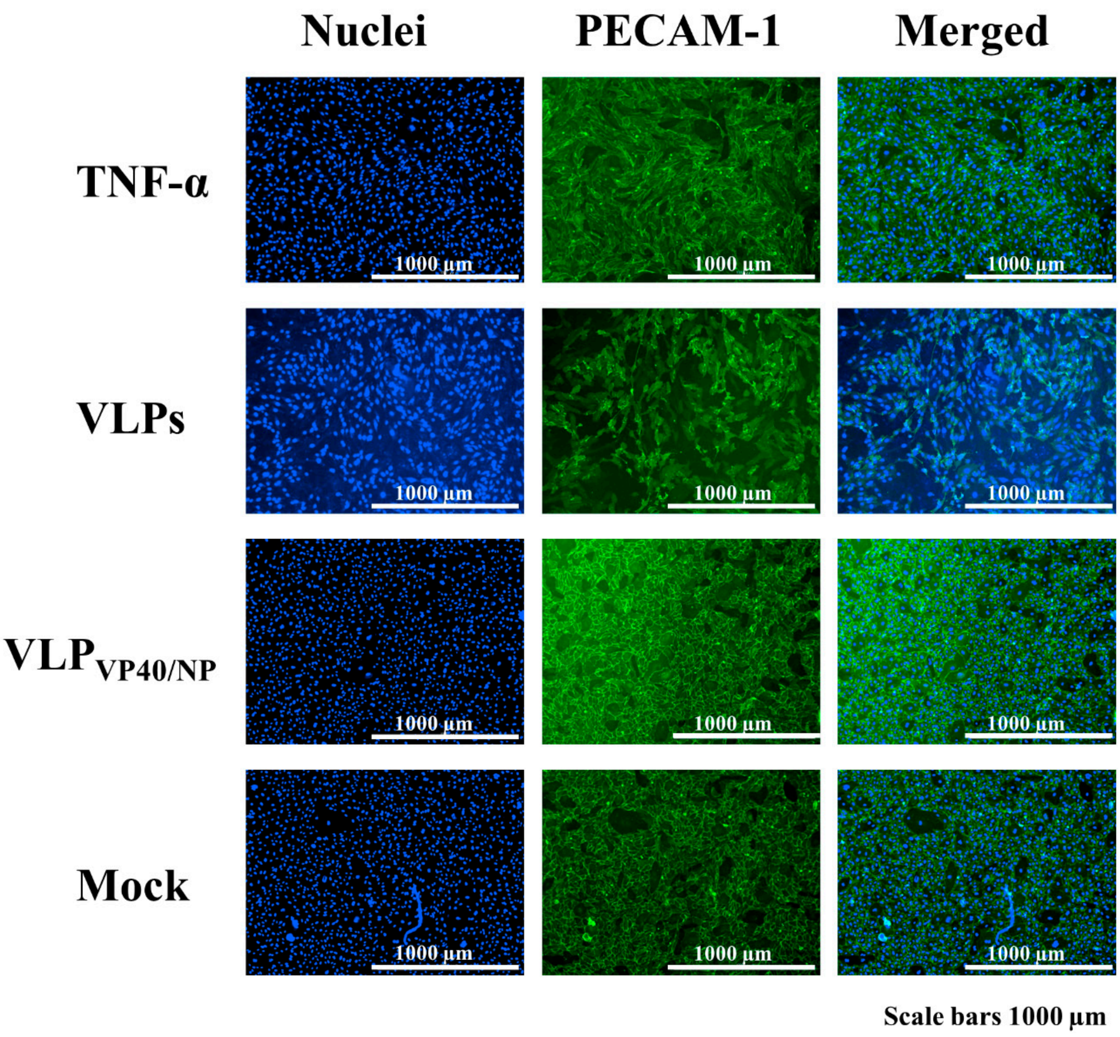

Figure S1. Platelet endothelial cell adhesion molecule 1 (PECAM-1) staining to confirm the integrity of the endothelial monolayer. Endothelial cells were exposed to Ebola Virus-like particles with GP (VLPs; 20× dilution) or without GP ($VLP_{VP40/NP}$; 20× dilution) for 48 h. Following the treatment, cells were fixed and permeabilized, and the activation was detected by immunofluorescence analysis using a monoclonal antibody directed against PECAM-1. The cells were then stained with Hoechst 33358 for imaging (magnification, ×10). Human recombinant TNF-α and supernatants from non-transfected cells (mock) were used as the positive and negative controls, respectively. Only cells treated with VLPs showed different pattern and shape for the PECAM-1 staining. TNF-α-, $VLP_{VP40/NP}^{-}$, and Mock-treated ECs showed similar patterns, confirming the integrity of ECs.

A

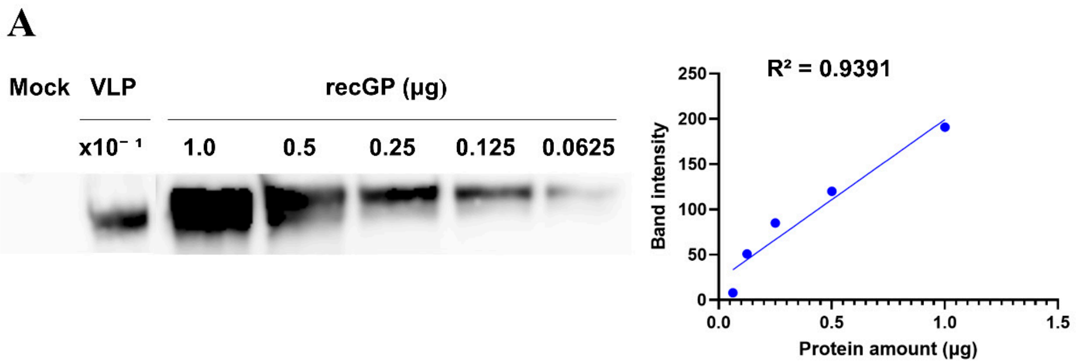

B

GP in VLP	Amount (μg)	Conc. (μg/ml)
x10^{-1}	0.4	9.2

Figure S2. Quantification of Ebola GP contained in the produced Ebola Virus-like particles (VLPs). (**A**) Western blot was performed with 10× dilution of Ebola VLPs and serial dilutions of the commercial recombinant GP lacking the transmembrane region (reference). Anti-GP antibodies were used to detect and analyze GP. The resulting linear standard curve (right panel) of the reference was used for the estimation of the amount of GP contained in VLPs. (**B**) the amount of GP contained in 10× dilution of VLPs was deduced from the linear curve equation.

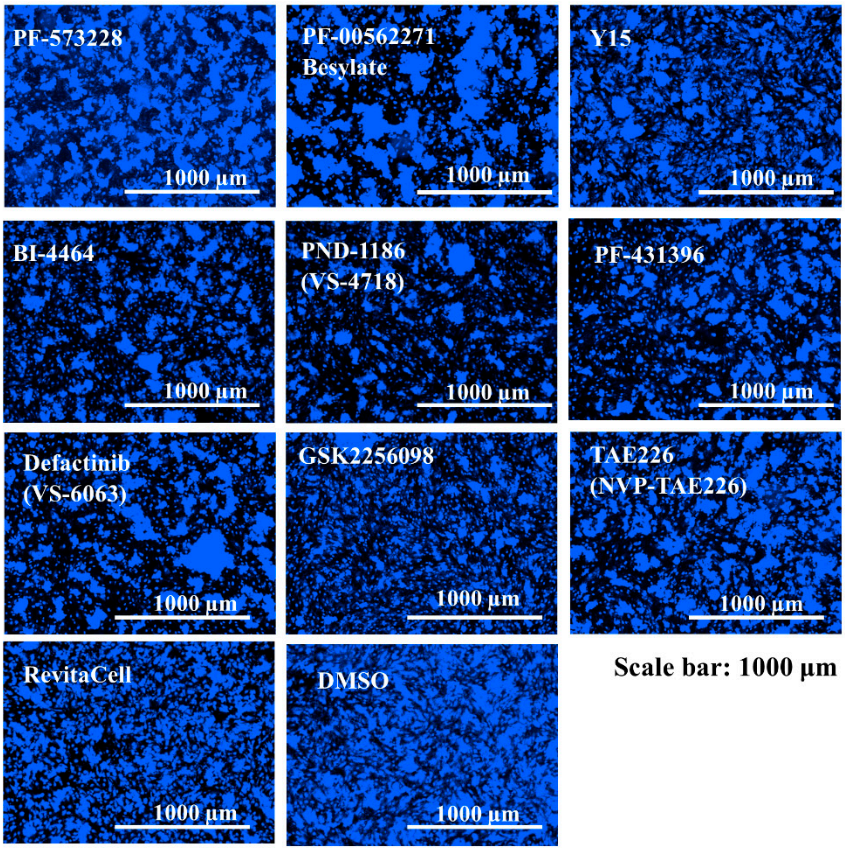

Figure S3. Nuclei staining of virus-like particles (VLPs)-treated endothelial cells in the presence of focal adhesion kinase (FAK) inhibitors. Endothelial cells in 96-well plates were treated with Ebola VLPs (10×) in the presence of FAK inhibitors (5 μM), with RevitaCell and DMSO used as the positive and negative controls, respectively. 48 hours post-treatment, the cells were analyzed by immunofluorescence for intercellular adhesion molecule 1 (data not shown) expression, and the nuclei (blue) were stained with Hoechst for the evaluation of the protective effect of FAK inhibitors against the VLPs-induced endothelial cells disruption.

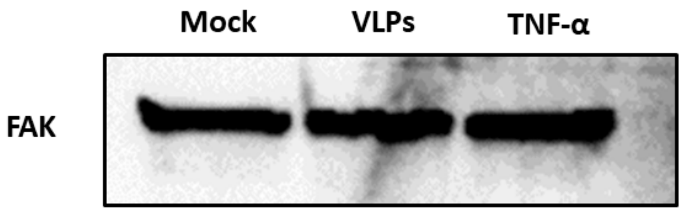

Figure S4. FAK detection in endothelial cells. Endothelial cells were treated with virus-like particle (10×) and TNF-α (10 ng/mL) for 48 h. Cell lysates were prepared and assessed for the level of focal adhesion kinase (FAK) expression using the rabbit monoclonal anti-FAK antibodies [(D507U)XP, Cell signaling, MA, USA; 1:1000]. Endothelial cells treated with the medium (Mock) were used as the control. No change in FAK level was observed upon the treatment with virus-like particles and TNF-α.

Table S1. Summary of cytoskeletal library screening for the inhibition of virus-like particles (VLPs)- and TNF-α-induced intercellular adhesion molecules 1 (ICAM-1) expression.

Compound Name	% of Inhibition of ICAM-1 Expression			
	VLPs-Induced ICAM-1		TNF-α-Induced ICAM-1	
	(Average)	+/-SD	(Average)	+/-SD
Imatinib Mesylate (STI571)	-41.7	12.6	3.3	4.1
Triciribine	-35.7	4.0	9.6	0.9
ABT-751 (E7010)	-41.1	19.6	4.5	9.7
Ispinesib (SB-715992)	-43.4	1.0	-7.6	14.3
SB743921 HCl	-64.6	37.6	-2.1	8.0
Quercetin	-33.0	48.8	10.7	0.6
A-674563	49.0	46.2	93.9	5.5
Ipatasertib (GDC-0068)	-50.4	43.5	2.4	1.3
Methyl Vanillate	-104.1	21.0	4.4	3.9
Vinorelbine Tartrate	45.2	29.4	34.2	1.4
Nilotinib (AMN-107)	-50.1	33.0	10.5	4.5
AT9283	82.8	17.0	91.4	2.8
BIIB021	74.8	9.7	32.8	0.4
Ponatinib (AP24534)	76.8	26.9	92.5	0.2
Lexibulin (CYT997)	10.3	27.7	23.8	0.6
Daphnetin	-0.3	17.1	-1.4	10.4
PF-00562271 Besylate	79.9	23.1	87.5	1.7
TAE226 (NVP-TAE226)	49.8	17.5	23.8	6.2
2-Methoxy-1,4-naphthoquinone	-87.7	0.2	26.9	38.5
Vinblastine sulfate	52.9	20.8	41.8	22.9
Elesclomol (STA-4783)	-24.8	6.5	-2.4	6.0
Tanespimycin (17-AAG)	73.1	14.3	30.2	3.2
XAV-939	-86.9	16.6	-8.3	0.7
PHT-427	-45.5	7.8	-10.2	3.1

NVP-BHG712	-7.4	6.8	-0.4	0.6
PP121	49.9	16.3	41.9	0.7
KW-2478	58.9	29.0	18.4	15.6
A-205804	54.7	4.6	33.3	11.5
Lifitegrast	-51.1	26.1	10.1	10.1
i-Inositol	-25.7	49.1	-1.6	5.9
Enzastaurin (LY317615)	-269.5	69.8	-11.7	5.0
Alvespimycin (17-DMAG) HCl	63.2	8.5	22.9	7.1
Vincristine sulfate	21.7	6.8	24.7	2.1
AT7867	23.2	5.6	-27.3	12.5
4-Demethylepipodophyllotoxin(NSC-122819,VM-26)	-40.7	21.7	10.4	8.5
Rebastinib (DCC-2036)	86.4	4.3	88.3	5.4
Geldanamycin	79.0	13.0	21.4	4.7
PF-562271	72.9	23.0	64.9	0.3
Decursinol angelate	-56.0	18.3	-2.2	18.8
Nilotinib hydrochloride	-26.8	15.4	4.0	1.8
Luminespib (AUY-922, NVP-AUY922)	100.3	8.4	51.0	37.1
Docetaxel	-18.8	9.3	13.2	7.5
Chelerythrine Chloride	-49.8	22.4	8.7	10.0
Albendazole	-62.5	12.0	-11.0	5.8
Colchicine	28.8	12.2	20.6	19.2
CCT128930	-29.7	22.9	-15.5	34.6
AZ 3146	-71.8	16.1	-16.7	8.9
GNF-2	-18.5	45.6	-3.2	10.2
Isoquercitrin	-8.9	17.6	-9.3	1.2
Dasatinib hydrochloride	95.6	5.8	-1.0	14.7
MK-2206 2HCl	-24.0	18.9	-3.9	11.4
Paclitaxel	-58.9	56.7	-9.1	33.6
Epothilone A	-17.6	5.9	-14.5	5.9
Albendazole Oxide	-86.9	31.2	-10.8	4.9
Honokiol	-39.5	49.7	23.2	50.2
SNX-2112 (PF-04928473)	51.3	2.9	15.7	33.4
PF-04691502	44.3	23.0	64.9	7.2
Go 6983	-38.8	45.9	-19.8	20.6
Vindoline	-14.7	9.9	-13.3	15.9
SC66	52.1	11.6	79.6	10.5
H-Cys(Trt)-OH	-28.5	3.6	-7.2	14.8
IWR-1-endo	-25.0	2.7	-2.5	23.6
Mdivi-1	-23.1	2.4	-8.2	14.7
IWP-L6	-64.4	66.9	-7.7	17.2
MPI-0479605	-59.8	32.3	-8.5	20.7
GNF-5	-79.9	45.6	-2.5	14.3
TRx0237 (LMTX) mesylate	-106.8	13.7	3.3	12.6
CP21R7 (CP21)	1.2	4.1	41.3	55.7
Wnt agonist 1	46.7	19.1	10.6	8.5
KYA1797K	-3.2	13.2	5.3	15.9
WAY-316606	-0.6	13.4	3.6	11.5
GSK923295	-31.2	7.9	-2.8	14.1
Dyngo-4a	-28.7	15.1	-2.2	17.8
CW069	-78.7	97.7	-4.9	17.7
WIKI4	-37.5	17.3	-3.6	11.6

SB273005	56.2	22.4	1.6	13.7
Akti-1/2	2.5	24.0	0.0	17.5
TIC10	-8.0	6.7	21.2	0.1
BAY 1217389	-44.0	15.6	3.3	5.4
Miransertib (ARQ 092) HCl	59.8	9.8	15.0	19.2
KHS101 hydrochloride	32.8	2.3	-0.5	12.2
IPA-3	-27.1	8.7	-6.8	16.6
GZD824 Dimesylate	83.2	3.6	94.4	0.0
CH5138303	82.2	14.0	17.8	8.0
Uprosertib (GSK2141795)	33.8	28.3	6.0	18.2
AT13148	23.2	33.7	-8.8	8.7
Dasatinib Monohydrate	58.7	13.7	-6.8	10.3
AZD5363	-11.6	42.7	-2.4	7.2
IQ-1	22.2	29.6	9.5	1.7
Apoptozole	-40.9	6.2	4.2	8.7
K 858	-0.5	29.0	-3.1	9.1
PF-3758309	77.0	17.9	91.4	0.8
Bisindolylmaleimide IX (Ro 31-8220 Mesylate)	71.9	9.7	96.6	2.0
ARQ 621	-9.8	46.8	-16.3	17.4
INH1	-178.7	239.4	-6.9	8.0
PF-431396	66.7	12.5	90.5	1.8
Combretastatin A4	24.7	10.9	0.5	8.5
PU-H71	63.8	9.4	25.0	7.7
PRI-724	-55.3	28.3	26.2	4.0
KRIBB11	28.4	9.5	38.1	4.4
BI-4464	78.2	6.3	13.2	3.0
KY02111	-46.8	16.1	-10.5	21.3
Bisindolylmaleimide I (GF109203X)	-60.1	33.4	-11.5	19.2
PF-562271 HCl	70.1	24.5	93.7	6.5
INH6	-25.5	30.1	3.4	14.7
PND-1186 (VS-4718)	40.2	34.0	21.5	14.7
Docetaxel Trihydrate	16.7	3.0	-1.8	18.2
Dynasore	-1.4	12.6	3.8	18.7
HA15	-56.7	18.9	-5.2	17.6
PNU-74654	-13.6	27.4	6.7	5.6
PKC-theta inhibitor	30.0	3.6	-3.9	15.0
XL888	89.7	1.0	15.3	14.3
PD173955	67.4	3.3	-12.1	28.7
VER-49009	68.4	4.0	-4.3	15.4
TAI-1	-31.9	28.3	-3.4	35.2
Ruboxistaurin (LY333531 HCl)	-52.6	29.1	-10.8	18.2
FRAX486	66.9	14.9	-9.3	21.1
Deguelin	-20.8	16.7	-6.2	13.7
TRC051384	53.2	11.7	12.7	13.1
Monastrol	-34.7	43.3	-0.8	15.3
LF3	17.9	11.3	1.7	2.6
GSK2256098	27.5	4.1	-3.5	2.5
Asciminib (ABL001)	-33.6	3.6	2.9	1.8
Tirofiban Hydrochloride	3.7	0.1	-3.8	1.4
GNF-6231	5.2	14.1	-0.3	8.6
IWP-O1	5.3	18.5	-5.0	1.2

iCRT3	-8.5	6.3	2.1	8.9
iCRT14	7.7	10.2	-1.8	0.2
Adavivint (SM04690)	-16.3	17.6	24.9	4.2
SSE15206	6.1	15.9	-0.4	1.9
Ingenol	8.4	17.3	10.6	3.2
Berbamine	41.9	7.3	3.7	7.4
Triptonide	88.8	19.3	99.1	0.5
Dasatinib	75.1	4.4	-1.2	0.4
GSK690693	-3.6	25.1	1.3	3.9
Onalespib (AT13387)	81.9	15.3	18.6	2.8
Bafetinib (INNO-406)	-12.7	29.8	-4.4	7.9
KW-2449	-31.0	8.0	9.8	2.7
ICG-001	-13.9	8.1	28.3	5.4
Sotrastaurin	-53.6	6.5	-15.2	5.4
Berbamine (dihydrochloride)	43.2	14.7	-1.8	3.8
Triclabendazole	8.6	15.6	-2.8	2.7
Danusertib (PHA-739358)	71.3	0.8	39.2	1.1
Ganetespib (STA-9090)	84.8	0.0	20.5	4.8
Patupilone (EPO906, Epothilone B)	15.3	3.9	0.1	2.4
PF-573228	50.7	10.9	1.1	3.8
Myricitrin	9.9	3.1	-0.2	5.0
PF-04929113 (SNX-5422)	75.7	3.6	21.4	3.8
Nocodazole	35.6	8.4	4.4	2.4
Cabazitaxel	31.1	11.4	0.7	1.5
Griseofulvin	28.9	1.1	-6.4	2.5
Y15	28.1	0.6	40.7	33.5
Trimethyloctadecylammonium bromide	25.3	16.5	12.1	1.7
Cilengitide?trifluoroacetate	34.1	2.1	4.2	2.2
LGK-974	25.1	2.9	9.6	5.9
NMS-E973	75.2	6.2	21.2	4.0
FH535	39.2	11.4	5.6	3.8
Afuresertib (GSK2110183)	44.6	5.4	-0.7	2.7
KNK437	17.2	7.7	7.9	5.6
Cyclo(RGDyK)	24.8	3.3	4.3	2.7
GNF-7	89.7	1.2	93.8	2.3
SKL2001	-55.0	22.9	-1.1	4.2
Wnt-C59 (C59)	-11.7	2.8	2.9	4.2
TIC10 Analogue	7.5	2.3	-1.2	6.5
FRAX597	26.0	34.8	-2.4	5.3
VER-50589	72.4	13.6	19.2	3.0
CK-636	1.9	1.9	-5.3	0.9
Monomethyl auristatin E (MMAE)	53.0	12.8	21.3	1.5
Cyclo (-RGDfK)	9.8	24.2	-1.6	2.0
Radotinib	-8.9	6.2	-3.9	1.7
Leukadherin-1	-0.6	31.1	-0.8	3.9
KPT-9274	23.1	15.7	-1.2	4.2
Perifosine (KRX-0401)	29.7	1.7	5.3	5.7
Miltefosine	22.9	0.1	11.1	9.2
Fosbretabulin (Combretastatin A4 Phosphate (CA4P)) Disodium	70.8	3.7	25.0	4.7
RGD (Arg-Gly-Asp) Peptides	-14.0	12.2	9.4	5.3

ATN-161 (Ac-PHSCN-NH2)	1.7	8.2	15.0	10.5
IWP-2	-22.2	34.1	2.3	5.5
Defactinib (VS-6063, PF-04554878)	80.3	21.9	84.7	11.3

Note: All compounds highlighted are FAK inhibitors selected as hit compounds because of their reduced cytotoxicity (Figure 6D & 6E). FAK: focal adhesion kinase.
